# Inhibition of NOX1/4 with GKT137831: a potential novel treatment to attenuate neuroglial cell inflammation in the retina

**DOI:** 10.1186/s12974-015-0363-z

**Published:** 2015-07-30

**Authors:** Devy Deliyanti, Jennifer L. Wilkinson-Berka

**Affiliations:** Department of Immunology, Monash University, Alfred Medical Research and Education Precinct Level 6, 89 Commercial Road, Melbourne, VIC Australia 3004

**Keywords:** Retinal ischemia, Inflammation, NADPH oxidase, Ganglion cell, Microglia, Müller cell

## Abstract

**Background:**

Inflammation and the excess production of reactive oxygen species (ROS) contribute significantly to the pathogenesis of ischemic retinopathies such as diabetic retinopathy and retinopathy of prematurity. We hypothesized that GKT137831, a dual inhibitor of NADPH oxidases (NOX) 1 and NOX4, reduces inflammation in the ischemic retina by dampening the pro-inflammatory phenotype of retinal immune cells as well as macroglial Müller cells and neurons.

**Methods:**

Ischemic retinopathy was induced in Sprague-Dawley rats by exposure to 80 % O_2_ cycled with 21 % O_2_ for 3 h per day from postnatal day (P) 0 to P11, followed by room air (P12 to P18). GKT137831 was administered P12 to P18 (60 mg/kg, subcutaneous) and comparisons were to room air controls. Retinal inflammation was examined by measuring leukocyte adherence to the retinal vasculature, ionized calcium-binding adaptor protein-1-positive microglia/macrophages, and the mRNA and protein levels of key inflammatory factors involved in retinal disease. Damage to Müller cells was evaluated by quantitating glial fibrillary acidic protein-positive cells and vascular leakage with an albumin ELISA. To verify the anti-inflammatory actions of GKT137831 on glia and neurons involved in ischemic retinopathy, primary cultures of rat retinal microglia, Müller cells, and ganglion cells were exposed to the in vitro counterpart of ischemia, hypoxia (0.5 %), and treated with GKT137831 for up to 72 h. ROS levels were evaluated with dihydroethidium and the protein and gene expression of inflammatory factors with quantitative PCR, ELISA, and a protein cytokine array.

**Results:**

In the ischemic retina, GKT137831 reduced the increased leukocyte adherence to the vasculature, the pro-inflammatory phenotype of microglia and macroglia, the increased gene and protein expression of vascular endothelial growth factor, monocyte chemoattractant protein-1, and leukocyte adhesion molecules as well as vascular leakage. In all cultured cell types, GKT137831 reduced the hypoxia-induced increase in ROS levels and protein expression of various inflammatory mediators.

**Conclusions:**

NOX1/4 enzyme inhibition with GKT137831 has potent anti-inflammatory effects in the retina, indicating its potential as a treatment for a variety of vision-threatening retinopathies.

## Introduction

Ischemic retinopathies such as retinopathy of prematurity and diabetic retinopathy are major causes of vision loss and blindness in infants and people of working age, respectively [1, 2]. Current treatments such as laser photocoagulation are used to reduce the severe microvascular and tissue damage that occur in the end stages of these diseases. However, although these treatments are somewhat successful, they do not prevent the advancement of retinopathy from its early to late stages. New strategies that target the underlying mechanisms involved in the development of ischemic retinopathies are of considerable interest.

Inflammation has emerged as an important factor in the vascular damage that develops in ischemic retinopathies as well as other retinal diseases and involves contributions from circulating leukocytes as well as a local neuroglial inflammatory response [3, 4]. The activation and proliferation of resident macrophages, known as microglia, injure retinal blood vessels by their increased production of cytokines [5, 6], and macroglial Müller cells, which have an integral role in the maintenance of the blood-retinal barrier, become gliotic and exhibit a pro-inflammatory phenotype [4, 7, 8]. Moreover, ganglion cells are injured by the increased production of cytokines from activated microglia [9]. These glial and neuronal cell populations are also key producers of vascular endothelial growth factor (VEGF), a potent angiogenic and permeability factor in the ischemic retina [7, 10], which also has pro-inflammatory actions including the attraction of leukocytes to tissues [11].

A central mechanism mediating these inflammatory pathways is the excess production of reactive oxygen species (ROS), which promotes the stabilization of the transcription factor, hypoxic inducible factor-1α, to increase the expression of VEGF and chemoattractants such as monocyte chemoattractant protein-1 (MCP-1) [12–14]. Tissue inflammation is exacerbated by further increases in ROS production due to stimulation by cytokines and growth factors including interleukin (IL)-6 and tumor necrosis factor-α (TNFα) [14, 15]. There is considerable interest in the role of the nicotinamide adenine dinucleotide phosphate (NADPH) oxidase enzyme family as the source of excess ROS production in a wide range of diseases including retinopathy [14]. Indeed, pharmacological approaches to target NADPH oxidase have been the subject of substantial investigation in order to develop relevant and effective medical treatments [16]. It is now appreciated that previous approaches have largely used agents that are non-specific for NADPH oxidase, which may account for their failure in a clinical setting [16].

Identification that the NADPH oxidase catalytic unit, NOX, exists in seven isoforms (NOX1 to 5, DUOX1, DUOX2) has led to significant research to determine the role of individual NOX isoforms in a variety of diseases and whether their inhibition has therapeutic potential [16–20]. NOX1, 2, and 4 are implicated in the pathogenesis of ischemic retinopathies [21–24], and phagocytic NOX2 contributes to retinal inflammation [23, 25]. However, due to NOX2’s key role in defense against pathogens, complete blockade of NOX2 may not be entirely suitable as a treatment approach in humans [16, 26]. Interestingly, emerging evidence indicates that NOX1 and NOX4 have pro-inflammatory actions [19, 27, 28]. GKT137831, a member of the pyrazolopyridine dione family, is a small molecule that potently inhibits NOX1 and NOX4 (Ki in the range of 100–150 nM in cell-free assays of ROS production using membranes prepared from cells heterologously overexpressing specific NOX enzyme isoforms) [29]. The therapeutic potential of GKT137831 has been demonstrated in various animal models of disease [19, 29–32] and is currently being evaluated in the clinic [16]. However, whether the benefits of GKT137831 extend to the extensive retinal inflammation that develops in ischemic retinopathies has not been explored. Here, using a rat model of retinopathy of prematurity, known as oxygen-induced retinopathy (OIR), and in vitro cultures of retinal cells, we show that GKT137831 attenuates the ischemia/hypoxia-induced pro-inflammatory phenotype of glial and neuronal cells involved in ischemic retinopathy.

## Methods

### NOX1/4 inhibition

GKT137831 (Genkyotex SA, Geneva, Switzerland) is an efficient inhibitor of both NOX1 and NOX4 isoforms (Ki 100–150 nM and *E*_max_ >90 %). GKT137831 has substantially less activity towards the NOX2 isoform in cell-free assays (Ki 1500 nM and *E*_max_ 60–70 %) and is also a weak inhibitor of the NOX2 isoform in cell-free assays but does not significantly inhibit neutrophil oxidative burst at concentrations up to 100 μM and does not inhibit innate microbial bacterial killing in vitro or in vivo (when used at a concentration of up to 20 μM or administered at 100 mg/kg orally, respectively). Furthermore, GKT137831 has no scavenging nor antioxidant activity when tested at up to 100 μM and has an excellent specificity for NOX1 and NOX4 enzymes as shown in an extensive in vitro off-target pharmacological profiling on which included 170 different proteins including ROS producing and redox-sensitive enzymes [30, 31].

### Animals

All procedures were approved by the Alfred Medical Research and Education Precinct (AMREP) Animal Ethics Committee (application E/1061/2011/M) and performed according to the National Health and Medical Research Council (NHMRC) of Australia’s Guidelines for the Care of Animals in Scientific Research. Sprague-Dawley rats were purchased from the Animal Resources Centre (ARC, Perth, Western Australia).

### Oxygen-induced retinopathy

OIR was induced in Sprague-Dawley rats using a previously published method [33–35]. OIR develops over two phases comprising vaso-obliteration (phase I) and neovascularization (phase II). In phase I, rats were exposed to 80 % O_2_ cycled with 21 % O_2_ for 3 h per day between postnatal days 0 and 11. Phase II of OIR occurred when rats were placed in room air between postnatal days 12 to 18. Controls were in room air for the entire study. Litters were randomized to receive a subcutaneous injection (100 μl) of GKT137831 (60 mg/kg/day) or vehicle (25 % dimethyl sulfoxide) between P12 to P18. Rat pups were entered into the study if they had a body weight of approximately 8 g on postnatal day 3. The body weight of pups was measured each day, and litter sizes were kept to 14 pups. The dose of GKT137831 was based on previous studies in which it effectively reduced vascular disease [33]. Rats were killed at postnatal day 18 with sodium pentobarbitone (170 mg/ml, Virbac, NSW, Australia).

### Immunohistochemistry for microglia and Müller cell gliosis in OIR

Using an established method [33, 35], 3-μm paraffin sections were incubated overnight at 4 °C with antibodies to ionized calcium-binding adapter molecule 1 (Iba1) to detect microglia (1:1000, Wako, Tokyo, Japan) and glial fibrillary acidic protein to detect gliosis (GFAP, 1:500, DakoCytomation, Glostrup, Denmark). A negative control without the primary antibody and an isotype IgG control was included in each experiment. Immunolabeling in sections incubated with Iba1 was visualized with a Vectastain ABC standard kit (Vector Laboratories Inc., CA, USA) and liquid DAB + substrate chromagen system (DakoCytomation). Immunolabeling for GFAP was visualized following incubation of the sections with Alexa Fluro 488-conjugated goat anti-rabbit IgG (1:200, Life Technologies, VIC, Australia). For quantitation, four sections at least 60 μm apart were randomly selected from each eye. In each section, four non-overlapping fields spanning the entire retina were captured at ×400 magnification using a Spot digital camera (SciTech, VIC, Australia). Image J software was used to set a threshold for immunolabeling which was applied to all fields. For Iba1, immunolabeling was quantitated in the inner retina (from the retinal surface to the inner plexiform layer), and for GFAP, all retinal layers were sampled. Five to six rats per group were evaluated.

### Retinal leukostasis

Following a previously published method [33, 36], rats were perfused via the right atrium with 0.1 M phosphate-buffered saline, pH 7.4, to clear blood cells and then rhodamine-conjugated Concanavalin A lectin (0.25 mg/kg, Vector Laboratories) to stain adherent leukocytes and the endothelium. Eyes were fixed in 4 % paraformaldehyde in 0.1 M phosphate-buffered saline, pH 7.4, for 30 min and retina flat-mounted. Non-overlapping images at ×200 magnification were captured, and the number of leukocytes per retina were counted. Six to nine rats per group were evaluated.

### Retinal vascular leakage

Albumin levels in freshly frozen single retina were measured according to the manufacturer’s instructions using a commercially available rat albumin ELISA quantification set (Bethyl Laboratories, Montgomery, TX, USA) as previously described [37]. Albumin values were normalized to dry retinal weight. Six to seven rats per group were evaluated.

### Protein levels of VEGF, MCP-1, and intercellular adhesion molecule-1 (ICAM-1) in retina

VEGF and MCP-1 were measured as described previously [37]. Briefly, supernatants from retinal lysates were collected, and the total protein concentration was quantitated using the Bradford colorimetric method (Bio-Rad Laboratories, NSW, Australia) according to the manufacturer’s protocol. Undiluted supernatants were then assayed using a commercially available rat VEGF, ICAM-1 (R&D Systems, MN, USA), and MCP-1 ELISA kits (BD Biosciences, CA, USA). All procedures were performed according to the manufacturer’s instructions. Six rats per group were evaluated.

### Real-time PCR

Total RNA was isolated using an RNeasy kit (Qiagen, Doncaster, VIC, Australia) according to the manufacturer’s protocol, and then 1 μg of RNA was subjected to DNase treatment (DNA-free kit, Ambion, Carlsbad, CA, USA) and reverse transcription (First Strand cDNA synthesis kit, Roche, Switzerland). For quantitative assessment of mRNA expression, gene-specific primers and cDNA (20 ng) were mixed with the SYBR Master Mix (Invitrogen), and real-time PCR was conducted using an ABI 7900 HT Sequence Detection System (Applied Biosystems) [34]. mRNA expression was normalized to 18s rRNA endogenous control, and the relative fold difference in expression was calculated using the comparative 2^-ΔΔCt^ method [34]. The primer sequences used in the study are previously published [37], except for platelet endothelial cell adhesion molecule-1 (PECAM-1), forward primer—5′GGCCAGCAGTCCCACTTCT3′ and reverse primer—5′TGTGTACTCCACATCCATGTTCTG3′. Six to ten rats per group were evaluated.

### Primary culture of rat retinal microglia and Müller cells

Primary cultures were performed as previously published [33, 35, 37]. Briefly, retinas were collected from 4- to 6-day-old Sprague-Dawley rats, digested, suspended in culture medium (low-glucose DMEM, Gibco, NY, USA), and cultured until reaching confluence at 10 to 12 days. Flasks were then shaken for 3 h at 37 °C at 200 rpm to remove microglia. After shaking, the culture supernatant was removed, and remaining cells were plated onto either poly-D-lysine coated 6-well plates or coverslips. Microglia and Müller cells were characterized as previously described [33, 35, 37]. Cells were exposed to hypoxia (0.5 % O_2_, 94.5 % N_2_, and 5 % CO_2_) in a modular incubator chamber (MIC101; QNA International Pty Ltd, VIC, Australia) for 4, 8, 16, or 72 h. Comparisons were made to cells exposed to normoxia. Experiments were repeated three to four times with at least three replicates per experiment.

### Primary culture of rat retinal ganglion cells

Primary cultures were performed as previously described with some minor modifications [35, 38]. Retinas were collected from 3-day-old Sprague-Dawley rats and digested for 10 min at 37 °C in Hanks balanced salt solution (HBSS, no Ca^2+^/Mg^2+^, Gibco) with 0.2 g/L trypsin and 80 U/ml DNase I. Digestion was quenched by the addition of 0.5 mg/ml soybean trypsin inhibitor (Gibco) in HBSS and suspended in HBSS with 0.2 % bovine serum albumin (Sigma, MO, USA). The cell suspension was passed through a 40-μM nylon mesh (BD Biosciences, CA, USA) and transferred to the negative panning plate coated with BS-1 lectin (10 μg/ml, Sigma) and incubated for 30 min at room temperature to remove microglia. Cells were then transferred to the positive panning plate coated with a goat anti-mouse IgM (10 μg/ml, chain specific, Jackson ImmunoResearch, PA, USA) a mouse anti-rat Thy1.1 antibody (10 μg/ml, clone T11D7e, Serotec, Oxford, UK) and incubated for 45 min at room temperature. Non-adherent cells were washed thoroughly with HBSS. Bound cells were collected by trypsination (0.25 %, Gibco). Ganglion cells were plated onto poly-D-lysine (10 μg/ml, Sigma) and laminin (50 μg/ml, Sigma) coated coverslips or 24-well plates in retinal ganglion cell culture media (Neurobasal media A [Invitrogen, Carlsbad, CA, USA], 1× B27 supplement [Gibco], 2 mM glutamine, 10 % dialyzed FBS [Gibco], 100 μM forskolin, 25 ng/ml ciliary neurotrophic factor, 10 ng/ml brain-derived neurotrophic factor, and 1× penicillin-streptomycin solution [Sigma]). Experiments were conducted 7 days after isolation. Characterization of retinal ganglion cells was as previously described [35]. GKT137831 was dissolved in vehicle (0.0001 % dimethyl sulfoxide). Ganglion cells were exposed to hypoxia (0.5 % O_2_, 94.5 % N_2_, and 5 % CO_2_) in a modular incubator chamber for 4 and 16 h. Comparisons were made to cells exposed to normoxia. Experiments were repeated three to four times with three replicates per experiment.

### ROS levels in rat retinal microglia, Müller cells, and ganglion cells

The levels of ROS were measured as previously reported [33]. Primary cultured cells were washed with HBSS for 2 min followed by a 30-min incubation with 5 μM dihydroethidium at 37 °C in 5 % CO_2_ in the dark. After incubation, cells were washed with HBSS and then imaged immediately using an Eclipse TE2000 inverted fluorescence microscope (Nikon Instruments Inc., Melville, NY, USA) and image processing computer software (NIS-Elements version 2.20, Nikon Instruments Inc.). The fluorescence intensity of cells was used as a measure of intracellular ROS levels using Image J analytical software (version 1.44, Bethesda, MD, USA). For quantitation, a minimum of 15 randomly selected cells from three different fields from each dish was evaluated. The intensity of fluorescence in a blank space between two cells was measured as background intensity. Three to four independent experiments were performed in each group with a minimum of three replicates.

### Protein levels of inflammatory mediators in cultured retinal cells

The protein levels of cytokines in supernatant of cultured rat microglia were measured using a rat cytokine antibody array performed according to the manufacturer’s instructions (R&D Systems Inc., Australia). The protein levels of MCP-1, VEGF, and soluble ICAM-1 (BD Biosciences, San Jose, CA, USA) as well as IL-6 (R&D Systems Inc.) were measured in supernatant of cultured rat Müller cells, and VEGF was measured in cultured rat ganglion cells using commercially available ELISA kits.

### Statistics

All data were analyzed using the GraphPad Prism Software (v.5, San Diego, California, USA). Normality was assessed by the Pearson, Shapiro-Wilk, and Kolmogoro-Smirnov normality tests. Analyses were performed by using a one-way ANOVA followed by appropriate post hoc analysis correcting for the number of comparisons and unpaired *t* tests (parametric) or a Kruskal-Wallis test followed by Mann-Whitney *U* tests (non-parametric). Investigators were masked to the groups. A value of *P* < 0.05 was considered significant.

## Results

### GKT137831 reduced Iba1-positive microglia and leukostasis in OIR

**Fig. 1:**
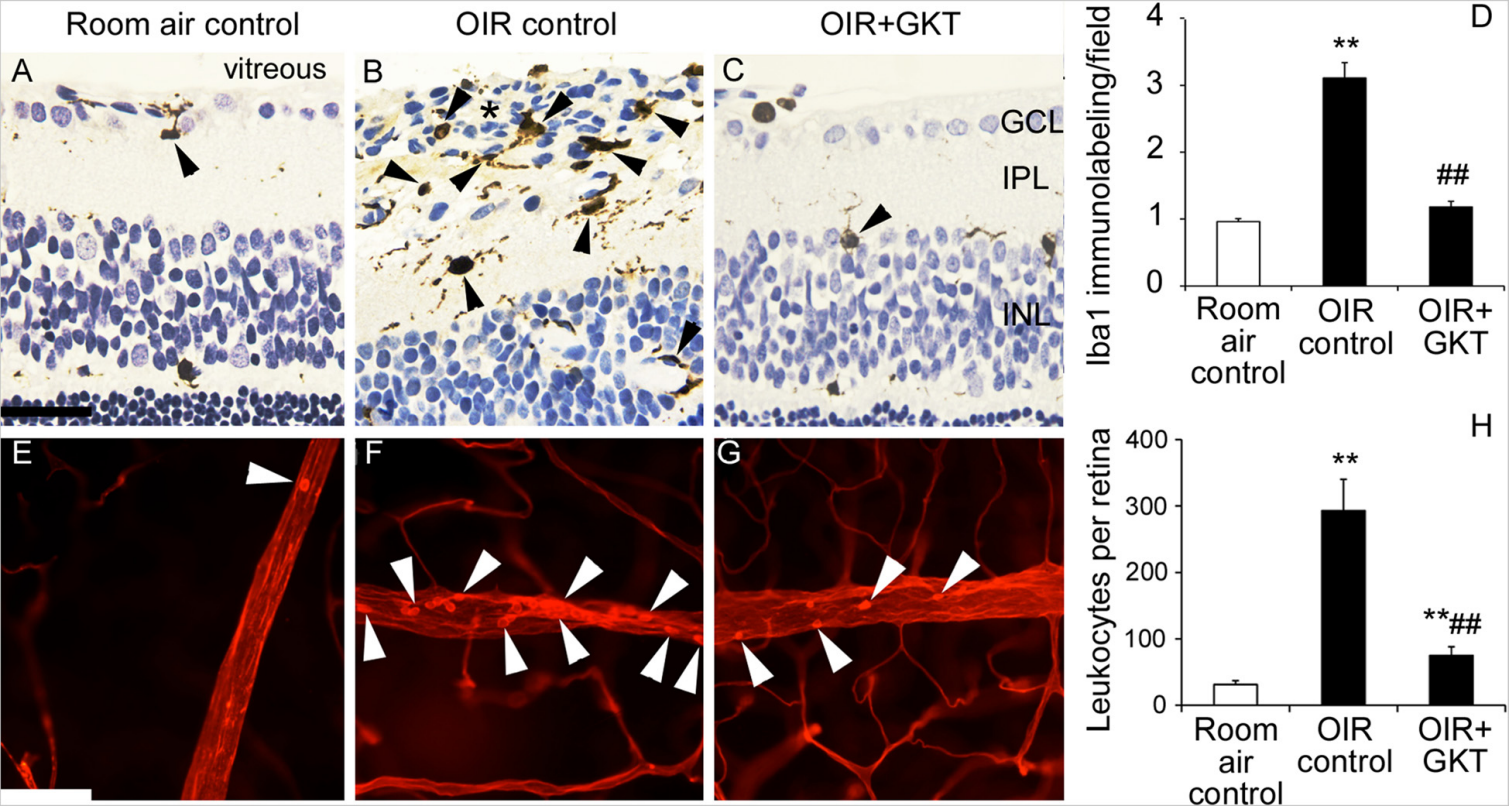
In OIR, GKT137831 reduced Iba1 immunolabeling of microglia and leukostasis. GKT137831 (GKT). **a**–**c** Representative 3-μm paraffin sections of retina immunolabeled with ionized calcium-binding adapter molecule 1 (Iba1, *arrowheads*). Iba1 immunolabeling is increased in OIR controls compared to room air controls, and in OIR controls, it is present in pre-retinal blood vessels (*asterisk*) protruding into the vitreous cavity. In OIR, treatment with GKT reduced Iba1 immunolabeling. Scale bar = 40 μm. *GCL* ganglion cell layer, *IPL* inner plexiform layer, *INL* inner nuclear layer. **d** Iba1 immunolabeling was quantitated in the inner retina (from retinal surface to IPL). *n* = 5 to 6 rats per group. **e**–**g** Representative whole mounts of retina from rats perfused with Concanavalin A to show leukocytes (*arrowheads*) adherent to the retinal vasculature. Leukostasis is increased in OIR controls compared to room air controls. In OIR, treatment with GKT reduced leukostasis. Scale bar = 40 μm. **h** Leukostasis was quantitated in the entire retina. *n* = 6 to 9 rats per group. **P < 0.01 to room air control. ##*P* < 0.01 to OIR control. Values are mean ± SEM

OIR is associated with increased microglial cell density in the retina compared to room air controls, which can be demonstrated by immunolabeling for Iba1 [33, 37]. This occurred in the present study with microglia in OIR control rats often localized to blood vessels protruding into the vitreous cavity (Fig. 1a to d). In OIR rats, GKT137831 reduced Iba1 immunolabeling to the level of room air controls (Fig. 1a to d). As the adherence of leukocytes to the retinal vasculature contributes to the development of ischemic retinopathy [11], we evaluated retinal leukostasis in rats with OIR. In OIR controls, retinal leukostasis was increased compared to room air controls. In OIR rats, GKT137831 reduced retinal leukostasis (Fig. 1e to h).

### GKT137831 reduced the expression of pro-inflammatory mediators in OIR

**Fig. 2:**
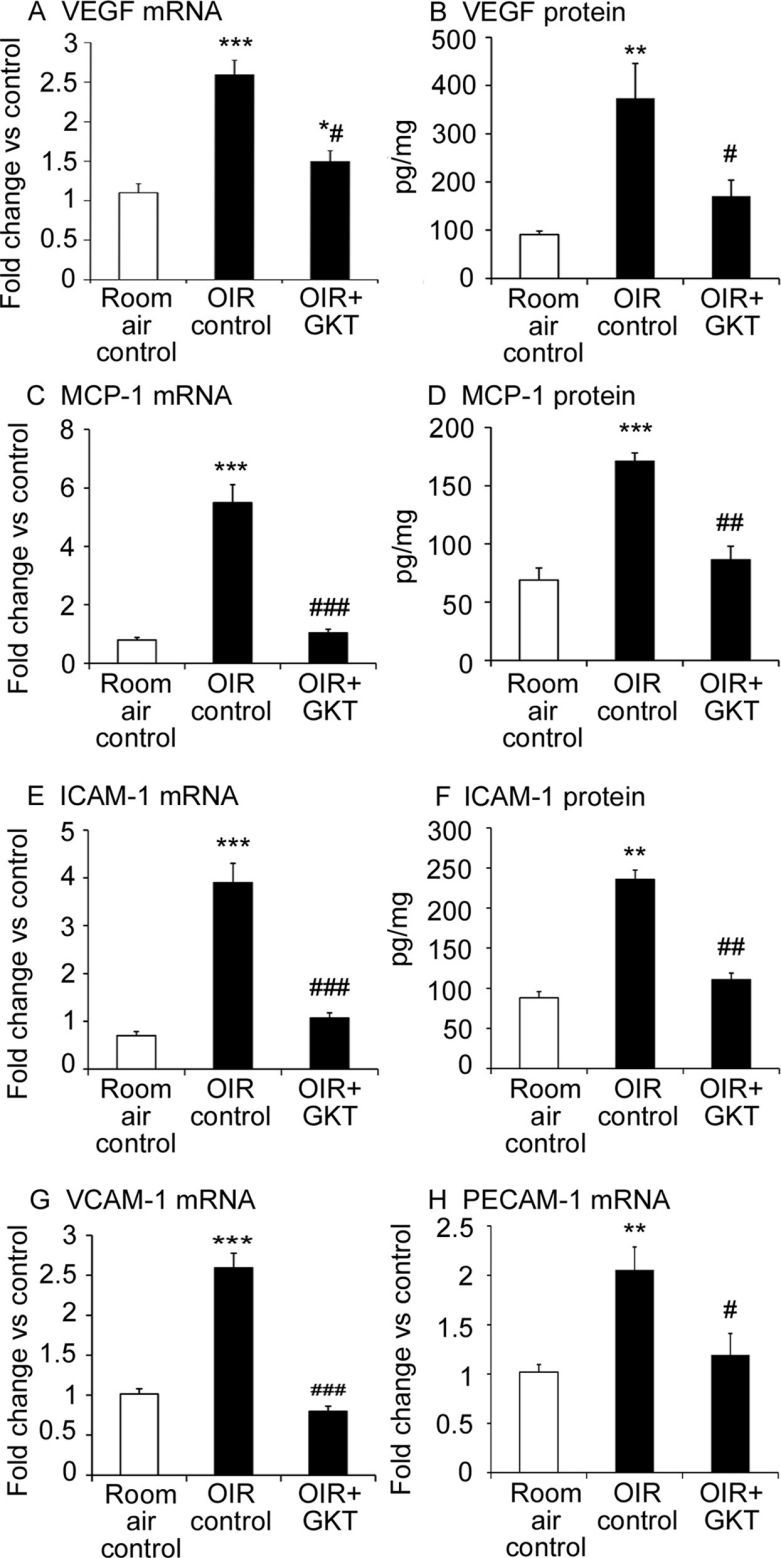
In OIR, GKT137831 reduced inflammatory mediators in the retina. GKT137831 (GKT). mRNA levels were determined in the whole retina by quantitative PCR. Protein levels in the whole retina were measured by ELISA. **a**, **b** VEGF mRNA and protein. **c**, **d** MCP-1 mRNA and protein. **e**, **f** ICAM-1 mRNA and protein. **g** VCAM-1 mRNA. **h** PECAM-1 mRNA. *P < 0.05, **P < 0.01, and ***P < 0.001 to room air control. #*P <* 0.05, ##*P* < 0.01, and ###*P* < 0.001 to OIR control. *n* = 9 to 10 rats per group. Values are mean ± SEM

As the upregulation of pro-inflammatory mediators is a feature of OIR, these factors were measured in the retina [33, 37]. OIR resulted in increased mRNA and protein levels of VEGF, MCP-1, and ICAM-1, as well as mRNA levels of vascular cellular adhesion molecule-1 (VCAM-1) and PECAM-1 compared to room air controls (Fig. 2a to h). In OIR rats, GKT137831 reduced all inflammatory factors (Fig. 2a to h).

### GKT137831 reduced retinal gliosis and vascular leakage in OIR

**Fig. 3:**
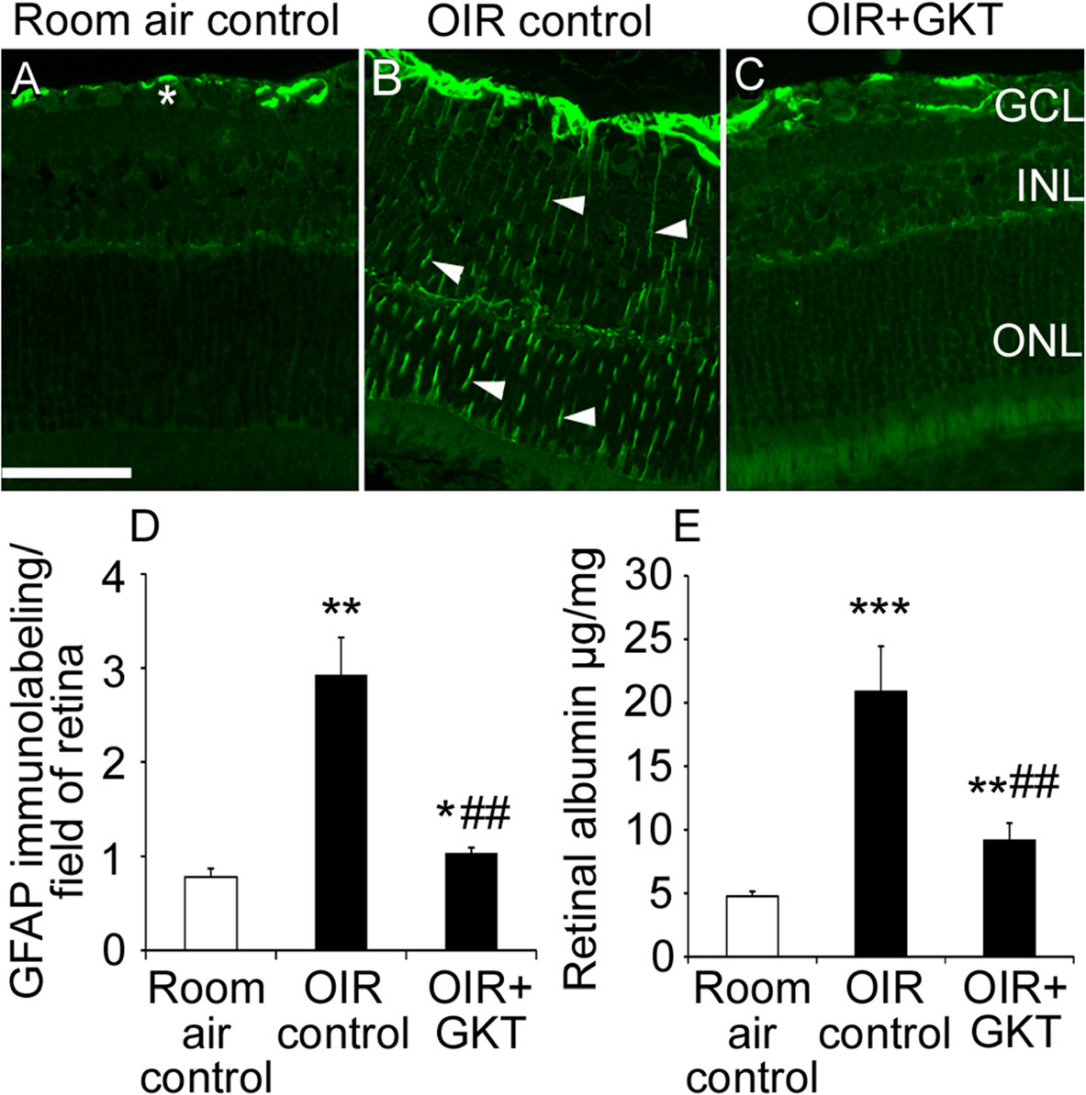
In OIR, GKT137831 reduced Müller cell gliosis and vascular leakage in retina. GKT137831 (GKT). **a**–**c** Representative 3-μm paraffin sections of retina immunolabeled with glial fibrillary acidic protein (GFAP). *GCL* ganglion cell layer, *INL* inner nuclear layer, *ONL* outer nuclear layer. In room air controls, GFAP immunolabeling is detected at the retinal surface (*asterisk*), whilst in OIR controls, GFAP immunolabeling extends throughout the retina in Müller cell processes (*arrowheads*). In OIR rats treated with GKT137831, GFAP immunolabeling is reduced compared to OIR controls. Scale bar = 40 μm. **d** GFAP immunolabeling was quantitated in the entire retina. *n* = 5 to 6 rats per group. **e** Vascular leakage was measured in single retina by an albumin ELISA. *n* = 6 to 7 rats per group. *P < 0.05, **P < 0.01, and ***P < 0.001 to room air control. ##*P* < 0.01 to OIR control. Values are mean ± SEM

Retinal gliosis can be robustly measured with immunolabeling for GFAP [4]. In room air controls, GFAP immunolabeling was restricted to the retinal surface (Fig. 3a), whilst in OIR controls, GFAP immunolabeling was present in Müller cell processes extending the width of the retina (Fig. 3b). In OIR rats, GKT137831 reduced GFAP labeling (Fig. 3c and d). As Müller cell gliosis is associated with disruption of the blood-retinal barrier [4], vascular leakage was measured. In OIR controls, vascular leakage was increased compared to controls. In OIR rats treated with GKT137831, vascular leakage was reduced compared to OIR controls (Fig. 3e).

### GKT137831 reduced ROS levels and the expression of inflammatory mediators in retinal microglia exposed to hypoxia

**Fig. 4:**
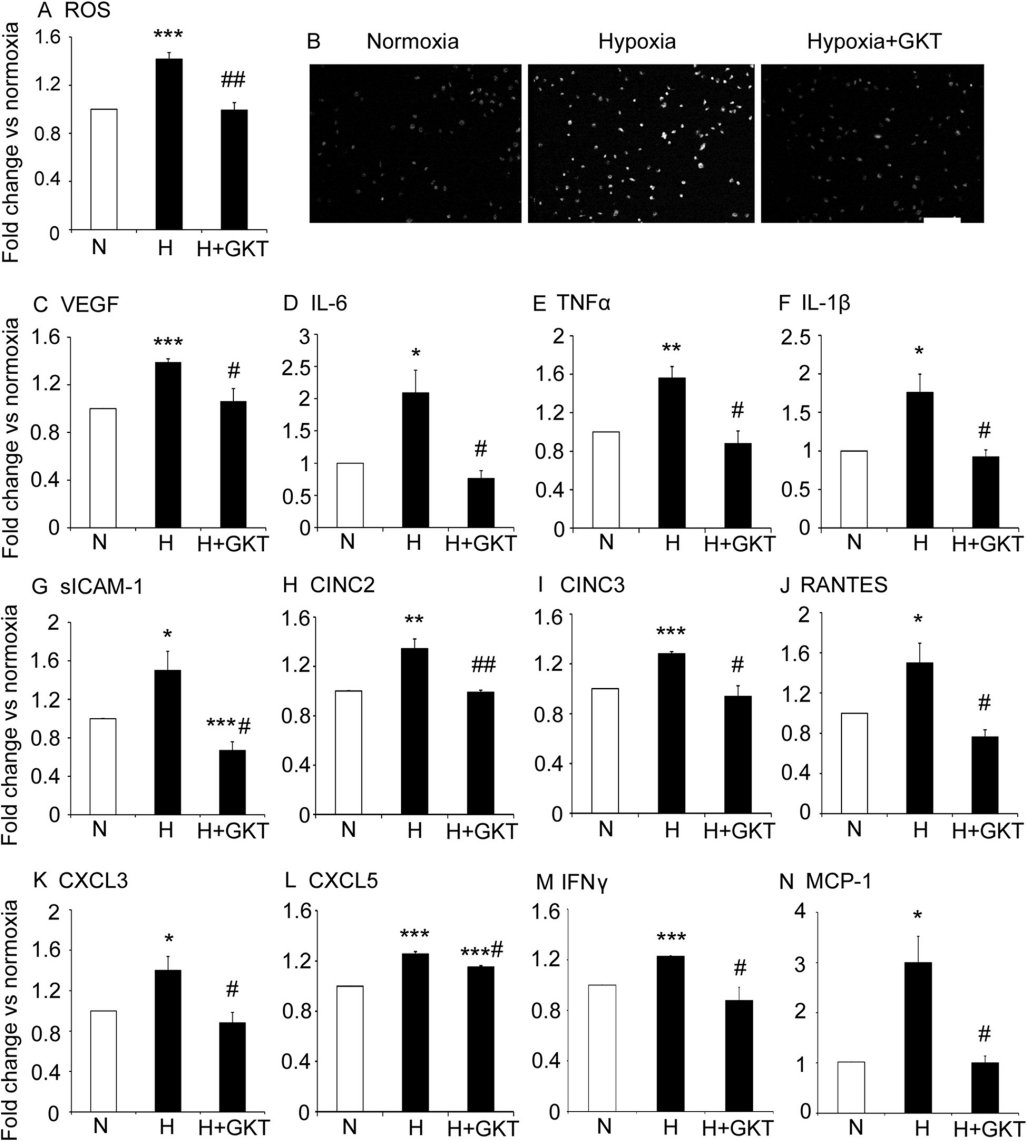
In cultured microglia, elevated ROS and protein levels of inflammatory mediators were reduced with GKT137831. *N* normoxia control (21 % O_2_), *H* hypoxia control (0.5 % O_2_). GKT137831 (GKT), 5 μM. Primary cultures of rat retinal microglia exposed to normoxia, hypoxia, and GKT for 16 h. **a** Quantitation of ROS levels. **b** Micrographs of retinal microglia labeled with dihydroethidium to detect ROS. Scale bar = 100 μm. **c**–**n** Protein levels of inflammatory mediators were measured by a rat cytokine antibody array. **c** VEGF. **d** IL-6. **e** TNFα. **f** IL-1β. **g** Soluble ICAM-1 (sICAM-1). **h** CINC2. **i** CINC3. **j** RANTES. **k** CXCL3. **l** CXCL5. **m** IFN_ϒ_. **n** MCP-1. *P < 0.05, **P < 0.01, and ***P < 0.001 to normoxia control. #*P* < 0.05 and ##*P* < 0.01 to hypoxia control. Values are mean ± SEM. *n* = 3 to 4 independent experiments and performed in triplicate

As microglia are the principal resident inflammatory cells in the retina, we examined ROS levels and the expression of a various inflammatory mediators implicated in ischemic retinopathy. Exposure of microglia to hypoxia for 4 h resulted in increased mRNA levels of NOX1, NOX2, and NOX4, and by 16 h, NOX1 and NOX4 mRNA levels remained elevated (Table 1). Subsequent experiments were performed at 16 h to allow for maximal increases in protein expression. After 16 h of hypoxia, ROS levels were increased compared to normoxia controls (Fig. 4a and b). In addition, protein levels of VEGF, IL-6, TNFα, IL-1β, ICAM-1, CINC2, CINC3, RANTES (regulated on activation, normal T cell expressed and secreted), CXCL3, CXCL5, interferon _ϒ_ (IFN_ϒ_), and MCP-1 were increased by hypoxia compared to normoxia controls (Fig. 4c to n). The hypoxia-induced increase in ROS and inflammatory mediators was reduced with GKT137831 (Fig. 4a to n).

**Table 1:** mRNA levels of NOX1, NOX2, and NOX4 in primary cultures of rat retinal microglia, Müller cells, and ganglion cells exposed to normoxia and hypoxia

	Microglia		Müller cells		Ganglion cells	
	4 h	16 h	8 h	72 h	8 h	16 h
NOX1						
N	1.01 ± 0.01	1.01 ± 0.00	1.00 ± 0.00	1.00 ± 0.01	1.00 ± 0.00	1.00 ± 0.00
H	1.31 ± 0.06**	3.06 ± 0.28**	0.90 ± 0.13	1.63 ± 0.11**	2.90 ± 0.25*	2.45 ± 0.47*
NOX2						
N	1.00 ± 0.00	1.02 ± 0.02	1.04 ± 0.04	1.01 ± 0.01	1.02 ± 0.01	1.06 ± 0.04
H	2.15 ± 0.21**	0.93 ± 0.07	0.87 ± 0.11	1.16 ± 0.18	1.26 ± 0.52	1.12 ± 0.21
NOX4						
N	1.01 ± 0.01	1.01 ± 0.01	1.00 ± 0.00	1.00 ± 0.01	1.03 ± 0.02	1.01 ± 0.00
H	1.66 ± 0.11***	1.77 ± 0.04***	0.96 ± 0.04	0.70 ± 0.14	1.02 ± 0.10	1.01 ± 0.12

Values are mean ± SEM. *n* = 3 to 4 independent experiments and performed in triplicate

*N* normoxia (21 % O_2_), *H* hypoxia (0.5 % O_2_)

*P < 0.05, **P < 0.01, and ***P < 0.001 to normoxia control

### GKT137831 reduced ROS levels and the expression of inflammatory mediators in retinal Müller cells exposed to hypoxia

**Fig. 5:**
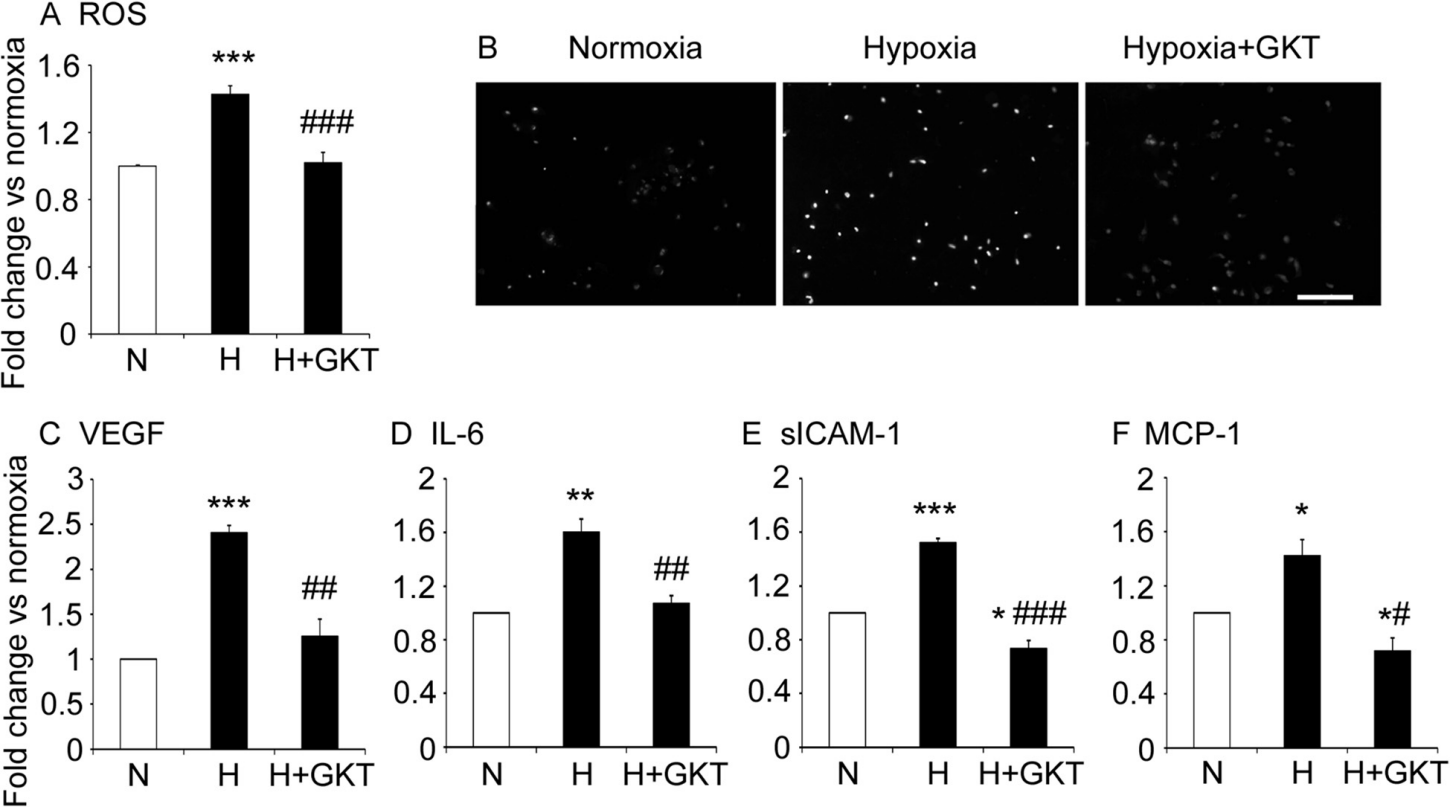
In cultured Müller cells, elevated ROS and protein levels of inflammatory mediators were reduced with GKT137831. *N* normoxia control (21 % O_2_), *H* hypoxia control (0.5 % O_2_). GKT137831 (GKT), 5 μM. Primary cultures of rat retinal Müller cells exposed to normoxia, hypoxia, and GKT for 72 h. **a** Quantitation of ROS levels. **b** Micrographs of retinal Müller cells labeled with dihydroethidium to detect ROS. Scale bar = 100 μm. **c**–**f** Protein levels measured by ELISA. **c** VEGF. **d** IL-6. **e** Soluble ICAM-1 (sICAM-1). **f** MCP-1. *P < 0.05, **P < 0.01, and ***P < 0.001 to normoxia control. #*P* < 0.05, ##*P* < 0.01, and ###*P* < 0.001 to hypoxia control. Values are mean ± SEM. *n* = 3 to 4 independent experiments and performed in triplicate

As macroglial Müller cells play an important role in retinal homeostasis [4], we examined ROS levels and the expression of inflammatory mediators known to be upregulated when Müller cells are injured [7, 39, 40]. Exposure of Müller cells to hypoxia for 8 and 72 h resulted in elevated levels of NOX1 mRNA but not NOX2 mRNA and NOX4 mRNA (Table 1). Subsequent experiments were performed at 72 h, and hypoxia resulted in elevated ROS levels compared to normoxia controls (Fig. 5a and b). Hypoxia increased the protein levels of VEGF, IL-6, soluble ICAM-1, and MCP-1 compared to normoxia controls (Fig. 5c to f). The hypoxia-induced increase in ROS and inflammatory mediators was reduced with GKT137831 (Fig. 5a to f).

### GKT137831 reduced ROS levels and the expression of VEGF in retinal ganglion cells exposed to hypoxia

**Fig. 6:**
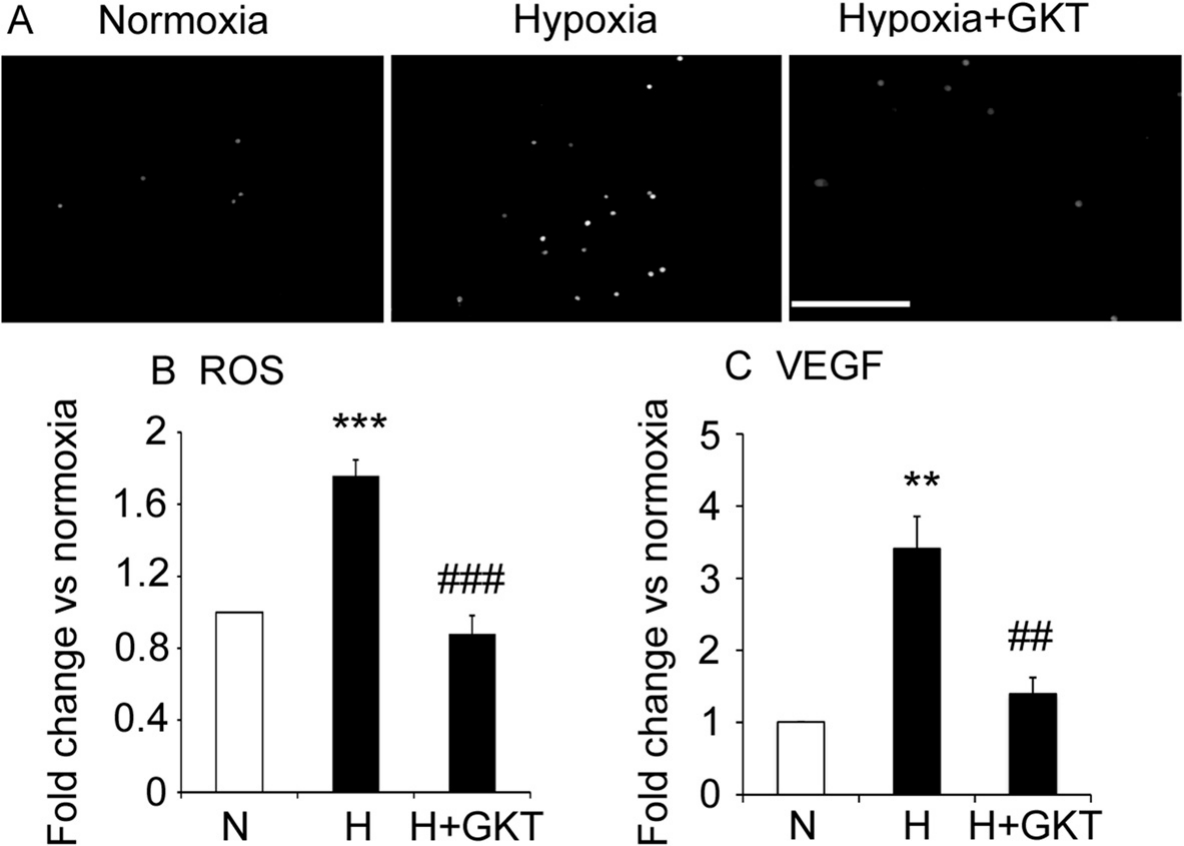
In cultured retinal ganglion cells, elevated ROS and VEGF protein levels were reduced with GKT137831. *N* normoxia control (21 % O_2_), *H* hypoxia control (0.5 % O_2_). GKT137831 (GKT), 5 nM. Primary cultures of rat retinal ganglion cells exposed to normoxia, hypoxia, and GKT for 16 h. **a** Micrographs of retinal ganglion cells labeled with dihydroethidium to detect ROS. Scale bar = 100 μm. **b** Quantitation of ROS levels. **c** VEGF protein levels measured by ELISA. **P < 0.01 and ***P < 0.001 to normoxia control. ##*P* < 0.01 and ###*P* < 0.001 to hypoxia control. Values are mean ± SEM. *n* = 3 to 4 independent experiments and performed in triplicate

Exposure of ganglion cells to hypoxia for 4 and 16 h resulted in elevated levels of NOX1 mRNA, but not NOX2 mRNA and NOX4 mRNA levels (Table 1). Subsequent experiments were performed at 16 h, and ROS levels and VEGF protein levels were increased by hypoxia compared to normoxia controls (Fig. 6a and c). The hypoxia-induced increase in ROS and VEGF were reduced with GKT137831 (Fig. 6c).

## Discussion

The chronic production of excessive ROS is central to the development of inflammatory diseases including those of the retina [14, 41]. Emerging evidence indicates ROS derived from NOX1, and NOX4 contributes to tissue inflammation [27, 28], and hence the development of specific inhibitors targeting these NOX isoforms is likely to have therapeutic potential for various inflammatory diseases. We previously reported dual NOX1/4 inhibition with GKT137831 reduced the OIR-induced increase in ROS levels and neovascularization in the retina [33]. The present study demonstrates for the first time that GKT137831 administered at the onset of tissue ischemia in phase II of OIR has marked anti-inflammatory actions, evidenced by a reduction in the adherence of circulating leukocytes to the retinal vasculature and the pro-inflammatory phenotype of retinal microglia, macroglial cells, and ganglion cells. Our in vitro studies, designed to resemble phase II of OIR, show that at the time points studied, hypoxia induced the upregulation of NOX1 and/or NOX4, and GKT137831 has direct anti-inflammatory actions on neuroglial cell populations.

Increased leukocyte adherence to the retinal vasculature is a feature of ischemic retinopathies [11, 42]. Indeed, the expression of leukocyte adhesion molecules such as ICAM-1 and VCAM-1 are increased in models of OIR [42, 43], and chemoattractants such as RANTES are elevated in the vitreous fluid of children with retinopathy of prematurity [44]. Consistent with the notion of systemic inflammation contributing to OIR are findings that vitreal macrophages are attracted to, and participate in, retinal neovascularization [45]. The ability of GKT137831 to reduce retinal leukostasis in OIR as well as the expression of MCP-1, ICAM-1, VCAM-1, and PECAM-1 in the retina suggests that GKT137831 interrupts leukocyte adherence to the retinal vasculature. This idea is in agreement with evidence that NOX1 is involved in the cytokine-induced adhesion of monocytes to vascular smooth muscle cells [46].

The involvement of microglia in retinal inflammation is complex as this cell type transitions from a protective, ramified, and M2 phenotype to an activated and amoeboid-shaped cell with M1 properties [47]. In an activated state due to hypoxia or other stressors, microglia are a major source of chemokines, adhesion molecules, and cytokines as well as growth factors, which have detrimental effects on various cell types in the retina [6]. Systemic inflammation may contribute with evidence that lipopolysaccharide administration to mice resulted in the activation of retinal microglia [48]. In the present study, GKT137831’s reduction of Iba1-positive microglia in OIR suggested a protective effect on this cell population due to inhibition of ROS derived from NOX1/4. However, it is recognized that Iba1 also labels macrophages, and hence, this cell type may have contributed to our evaluation of retinal microglia. By utilizing primary cultures of retinal microglia exposed to hypoxia to mimic the ischemia that occurs in phase II of OIR, we were able to directly assess this cell population and show that NOX1, NOX4, and ROS levels are increased. The ability of GKT137831 to reduce the hypoxia-induced increase in ROS and the inflammatory molecules, VEGF, IL-6, and TNFα, indicates that dual inhibition of NOX1/4 has potent anti-inflammatory effects in the key immunocompetent cell of the retina. GKT137831’s anti-inflammatory properties extended to a reduction in leukocyte recruitment molecules including MCP-1, RANTES, CINC2, CINC3, CXCL3, and CXCL5 as well as ICAM-1 and VCAM-1 and emphasize the ability of this agent to dampen the strong pro-inflammatory signals produced by activated retinal microglia. Our findings are consistent with recent reports showing GKT137831 reduced macrophage infiltration in diabetic kidney [49] and T cell numbers in atherosclerosis [19]. A limitation of our study is that we did not evaluate whether inhibition of ROS derived from NOX1/4 in retinal microglia is the primary event conferring protection against hypoxia-mediated damage to other glia cell populations and neurons in the retina [9, 39]. Certainly, future studies in this aspect of retinal inflammation are of interest.

Macroglial Müller cells have numerous functions that are vital to retinal homeostasis including neuroprotection, suppression of inflammation, and maintenance of the blood-retinal barrier [4]. When injured, Müller cells exhibit a pro-inflammatory phenotype [4] reflected by increased expression of MCP-1 and ICAM-1 [50, 51]. The health of Müller cells is robustly demonstrated by measuring GFAP, a marker of intermediate filaments [4]. In uninjured retina, GFAP expression is restricted to astrocytes at the retinal surface, whilst in disease, Müller cells exhibit GFAP along their cell processes which span almost the entire width of the retina [4, 6, 8]. In OIR, Müller cell gliosis and inflammation is a major contributor to breakdown of the blood-retinal barrier [4, 52]. GKT137831’s anti-inflammatory effects in Müller cells suggest this translated to prevention of their gliosis and vascular leakage in OIR. Our findings in cultured Müller cells confirmed GKT137831’s anti-inflammatory properties, and the upregulation of NOX1 in response to hypoxia suggested that this NOX isoform is likely to be involved in ROS-mediated inflammatory effects in Müller cells.

An important finding was the ability of GKT137831 to reduce VEGF in microglia, Müller cells, and ganglion cells. VEGF’s potent pro-angiogenic and pro-permeability actions are well documented and form the basis of treatments such as bevacizumab for diabetic retinopathy [53]. Furthermore, VEGF is a potent chemoattractant stimulating increased ICAM-1 expression and leukocyte adhesion in the retina [11]. In a previous study, we demonstrated GKT137831 reduced retinal neovascularization in rats and mice with OIR [33] but did not evaluate in detail the retinal cell types in which GKT137831 modulated VEGF expression. Our findings in the current study indicate that GKT137831’s effects in ischemic retinopathy involve the attenuation of amplified VEGF levels in neuroglial cell populations. In addition, retinal ganglion cells, which make a major contribution to VEGF production in phase II of OIR [10], predominately express NOX1. This finding is consistent with a previous study showing that hypoxia and glucose deprivation induced the expression of this NOX isoform in retinal ganglion cells [54].

## Conclusions

Increasing evidence indicates that ROS derived from specific isoforms of NOX contributes to retinal disease. Here, we describe the ability of a specific NOX1/4 inhibitor, GKT137831, to attenuate ischemic-induced inflammation in the retina and neuroglia. GKT137831 is currently in clinical development, albeit for other diseases. Our findings suggest that NOX1/4 inhibition may provide a new treatment strategy for inflammatory ocular diseases, which feature the excess production of ROS from these NOX isoforms.

## Abbreviations

GFAP: glial fibrillary acidic protein
GKT: GKT137831
HBSS: Hanks balanced salt solution
Iba1: ionized calcium-binding adapter molecule 1
ICAM-1: intercellular adhesion molecule-1
IL: interleukin
MCP-1: monocyte chemoattractant protein-1
NADPH: nicotinamide adenine dinucleotide phosphate
OIR: oxygen-induced retinopathy
PECAM-1: platelet endothelial cell adhesion molecule-1
RANTES: regulated on activation normal T cell expressed
ROS: reactive oxygen species
TNFα: tumor necrosis factor-α
VCAM-1: vascular cellular adhesion molecule-1
VEGF: vascular endothelial growth factor
